# Efficacy, Safety and Tolerability of a New 10% Intravenous Immunoglobulin for the Treatment of Primary Immunodeficiencies

**DOI:** 10.3389/fimmu.2021.707463

**Published:** 2021-07-08

**Authors:** Elena E. Perez, Jacques Hébert, Anne K. Ellis, Oral Alpan, William R. Lumry, Ralph Shapiro, Daniel Suez, J. Fernando Mandujano, Richard L. Wasserman

**Affiliations:** ^1^ Allergy Associates of the Palm Beaches, North Palm Beach, FL, United States; ^2^ Department of Medicine, Laval University, Québec City, QC, Canada; ^3^ Division of Allergy and Immunology, Department of Medicine, Queen’s University, Kingston, ON, Canada; ^4^ Section on Immunopathogenesis, O&O Alpan, Fairfax, VA, United States; ^5^ Allergy and Asthma Research Associates Research Center, Dallas, TX, United States; ^6^ Immunology Department, Midwest Immunology Clinic, Plymouth, MN, United States; ^7^ Allergy, Asthma & Immunology Clinic, PA, Irving, TX, United States; ^8^ Pediatric Pulmonary Associates of North Texas, Frisco, TX, United States; ^9^ Allergy Partners of North Texas Research, Dallas, TX, United States

**Keywords:** primary immunodeficiency disease, inborn errors of immunity, immune globulin intravenous, intravenous immune globulin, immunoglobulin replacement therapy, IVIG, IgG, GC5107

## Abstract

We report here the results of a phase 3 study to assess the efficacy, safety, and tolerability of GC5107, a new 10% liquid intravenous immunoglobulin (IVIG) in preventing serious bacterial infections in patients with primary immunodeficiency (ClinicalTrials.gov: NCT02783482). Over a 12-month study period, 49 patients aged 3 to 70 years with a confirmed diagnosis of primary immunodeficiency received GC5107 at doses ranging from 319 to 881 mg/kg body weight every 21 or 28 days, according to their previous IVIG maintenance therapy. A total of 667 infusions of GC5107 were administered comprising a total of 45.86 patient-years of treatment. A single acute serious bacterial infection occurred during the study, resulting in an incidence of 0.02 events per patient-year (upper 99% one-sided confidence interval limit: 0.21), meeting the prespecified primary efficacy endpoint. The mean incidence of infections other than acute serious bacterial infections was 2.9 infections per patient-year. Efficacy was also demonstrated by the low mean annualized rate of hospitalizations due to infection (0.1 day) and the mean annualized duration of hospitalizations (0.1 day). The mean rate of intravenous and oral antibiotic use was 0.1 day and 13.2 days, respectively. There was a mean of 7.1 days of missed work, school, or daycare days. The proportion of infusions with temporally associated adverse events (TAAEs) occurring during or within 72 hours after GC5107 infusion was 0.24 (upper 95% one-sided confidence interval limit: 0.31), meeting the pre-specified primary safety endpoint. Overall, 149 of 667 infusions (22%) were associated with TAAEs. The most common TAAE was headache, reported by 49% of patients. More than 98% (731/743) of all adverse events that occurred throughout the 12-month study period were mild or moderate. More than 98% of infusions were completed without discontinuation, interruption or rate reduction. There were no treatment-emergent serious adverse events related to GC5107 or study discontinuations due to an adverse event. Overall, pharmacokinetic parameters for GC5107 were within the range of those reported in studies of other marketed IVIG products. Results of the present study demonstrate that GC5107 is an effective, safe and well-tolerated treatment for patients with primary immunodeficiency.

## Introduction

Primary immunodeficiency diseases (PIDs) comprise a large, heterogenous group of disorders resulting from inborn errors of immunity. Patients with PID are unable to mount an immune response to pathogens, and can experience recurrent bacterial, viral, fungal and protozoal infections as a result (1, 2). To date, over 250 PIDs have been described and over 450 genetic defects resulting in inborn errors of immunity have been characterized (3, 4). Rapid advances in genetic diagnosis increase the likelihood that this list will continue to expand (4, 5). Antibody deficiencies comprise the largest group of PIDs and are characterized by an impaired ability to produce specific antibodies in response to antigen (2). Patients with antibody deficiencies can present at any age.

Immunoglobulin (IG) replacement therapy has been standard of care for patients with antibody deficiencies for decades (3, 6). IG is formulated for either intravenous (IVIG) or subcutaneous (SCIG) administration. Because IG is purified by fractionation of large volumes of pooled human plasma from thousands of healthy donors, the supply is finite. Shortages have occurred in the past that have resulted in prioritization of patients, and changes in IG doses and infusion schedules (7, 8). The COVID-19 pandemic has introduced additional stress on plasma supply and plasma product demand (9), underscoring the importance of developing new IG products in order to increase therapeutic options for patients with PIDs for whom IG is a critical therapy.

GC5107 is a new 10% liquid IVIG product composed of human IG produced by GC Pharma (GCC) in Korea. GC5107 is manufactured from pooled human plasma from healthy US adult donors. The manufacturing process involves modified Cohn-Oncley cold ethanol fractionation and includes column chromatography (to reduce the content of thrombogenic impurities, such as coagulation factor XIa) (10), viral inactivation by solvent/detergent processing, and viral removal by fraction I+III precipitation and nanofiltration. Formulated as a solution, GC5107 contains 100 mg/mL immunoglobulin G (IgG) and 250 mM glycine, and has a pH of 4.8.

The current clinical study was undertaken to assess the clinical efficacy and safety of GC5107 in patients with PID.

## Materials and Methods

### Study Design

This was a phase 3, open-label, single-arm, historically controlled, multicenter study of GC5107 in patients with a confirmed diagnosis of PID as defined by the International Union of Immunological Societies criteria (1). All study sites (10 in the US and 7 in Canada) received Institutional Review Board/Research Ethics Board approval prior to enrolling subjects. The study was performed in accordance with the International Conference on Harmonization Good Clinical Practice Guidelines and in compliance with the regulations of the US Food and Drug Administration (FDA) and Health Canada. To participate in the study, all subjects (or parents or guardians of pediatric and adolescent subjects) provided written informed consent/assent. A third-party Drug Safety Monitoring Board, independent of both the sponsor and participating sites, monitored the safety of subjects throughout the study.

### Study Subjects

To be eligible for enrollment, patients between the ages of 2 and 70 years were required to have documented agammaglobulinemia or hypogammaglobulinemia. Study participants must have received 300-900 mg/kg of a licensed IVIG therapy at 21- or 28-day intervals for at least 3 infusions prior to study enrollment and have at least 2 documented trough IgG levels of ≥500 mg/dL within 12 months of enrollment.

Exclusion criteria included a history of a severe reaction or hypersensitivity to other IgG products, a history of thrombotic events, protein-losing enteropathy, nephrotic syndrome or lymphangiectasia, clinical signs or symptoms of an acute infection within 7 days prior to screening, a known history of or positive screening test for markers of infectious viruses, persistent severe neutropenia, and transaminases above 2.5 times the upper limit of normal. Females who were pregnant, nursing or planning a pregnancy during the study were excluded.

A subpopulation of enrolled subjects provided a separate informed consent in order to participate in the pharmacokinetic (PK) assessment.

### Study Medication

GC5107 (immune globulin intravenous [human]) is a 10% liquid IVIG manufactured from pooled human plasma from adult US donors. Viral inactivation and removal steps include solvent/detergent treatment, precipitation, and nanofiltration. GC5107 contains approximately 100 mg/mL IgG and 250 mM glycine, and has a pH of 4.8. GC5107 contains not less than 96% IgG monomers and dimers.

### Treatments

Over the 12-month study period, subjects received infusions of GC5107 at doses ranging from 319 to 881 mg/kg body weight every 21 or 28 days, according to their previous IVIG maintenance therapy. The dose and infusion interval remained unchanged throughout the study unless a change was medically justified. Subjects received a total of 17 infusions of GC5107 (for those on the 21-day schedule) or 13 infusions (for those on the 28-day schedule).

The first infusion of GC5107 was started at a rate of 1.0 mg/kg/min for 30 minutes. If well tolerated, the infusion rate was doubled every 30 minutes to a maximum of 8.0 mg/kg/min at 90 minutes and remained at that rate until the infusion was completed. If the first infusion was well tolerated, subsequent infusions were started at an initial rate of 2.0 mg/kg/min and increased every 15 minutes to a maximum of 8.0 mg/kg/min.

After completion of the last study infusion, subjects returned for a follow-up visit within 3 weeks (21-day schedule) or 4 weeks (28-day schedule). The total study period, including the treatment period and follow-up, was thus approximately 13 months.

### Efficacy Evaluation

The primary measure of efficacy was the incidence of acute serious bacterial infections (aSBIs) meeting FDA criteria, which include bacterial pneumonia, bacteremia/sepsis, bacterial meningitis, visceral abscesses, and osteomyelitis/septic arthritis.

Secondary efficacy outcome measures included the incidence of infections other than aSBIs; the number of days missed from work, school, or daycare or inability to perform normal activities due to an infection; the number of unscheduled physician visits and days of hospitalization due to an infection; days on therapeutic antibiotics (intravenous [IV] and oral); and the incidence of infections other than aSBIs correlated with trough IgG levels.

Information for secondary efficacy measures was collected in subject diaries, which were reviewed by study personnel at day 4 after the first 2 infusions of GC5107, at each treatment visit, and at the follow-up visit.

### Safety Evaluation

The primary safety endpoint was the proportion of infusions with TAAEs occurring during or within 72 hours following infusion, whether or not they were thought to be related to GC5107.

Safety assessments included physical examinations, vital signs, routine safety laboratory tests (hematology, blood chemistry, and urinalysis), direct Coombs test and viral safety testing. Subjects were instructed to record in their diaries any adverse events (AEs), particularly those that occurred within 24, 48, and 72 hours post-infusion, as well as any medications taken.

Secondary safety measures included the overall incidence of all TAAEs (whether or not they were determined to be related to GC5107); the frequency of all AEs occurring during the study; the frequency of suspected adverse reactions (ARs) (AEs determined to be at least possibly related to GC5107); the proportion of AEs considered by the investigators to be related to GC5107; the number and proportion of infusions during which the infusion rate was decreased due to AEs; and changes in vital signs, physical examination and laboratory results, and viral safety.

AEs were evaluated for severity (mild, moderate or severe), relationship to GC5107 (certain, probable, possible, or not related), seriousness (serious or non-serious), outcome (recovered/resolved or not recovered/not resolved) and temporal relationship with the infusion.

### Pharmacokinetic Evaluation

The primary PK objectives were to assess the PK parameters for total IgG (assessed in the PK population of 27 subjects) and trough serum total IgG levels prior to each infusion (assessed in all subjects).

Blood samples were collected from all subjects at screening, prior to each infusion of GC5107 and at the follow-up visit to determine total IgG serum trough levels. Patients in the PK subpopulation underwent additional blood sampling prior to the 5^th^ infusion, and at 30 minutes, 120 minutes, 24 hours, 48 hours, and at days 4, 8, 15, 22, and 29 following the 5^th^ infusion to determine total IgG PK parameters.

Secondary PK objectives included measurements of (1) the PK parameters of IgG subclasses (in the PK population), (2) trough serum levels of IgG subclasses and anti-*Haemophilus influenzae* type b, anti-*Streptococcus pneumoniae* serotypes, anti-tetanus toxoid, and anti-cytomegalovirus (CMV) antibodies at 4 time points throughout the study, and (3) the number and proportion of subjects who failed to meet the target IgG trough level (500 mg/dL) at any time at or after the 5^th^ infusion.

### Statistical Analyses

Primary safety and efficacy analyses were based on the intent-to-treat (ITT) population, consisting of all enrolled subjects who received any amount of GC5107. For some analyses, subjects receiving infusions every 3 weeks and every 4 weeks were considered both separately and as the total ITT population.

For the safety analysis, in order to estimate the overall probability of the occurrence of an AE possibly related to infusion for study subjects, as well as an upper 95% confidence interval (CI) limit, the Generalized Estimating Equation method of Zeger and Liang was used (11). Adverse events were mapped to a MedDRA version 22.0 preferred term and system organ class.

PK parameters were calculated by noncompartmental analysis methods from the concentration-time data using WinNonlin (WNL) Professional (Version 8.0 or higher). All statistical analyses were performed using Statistical Analysis System (SAS^®^) version 9.4 in a secure and validated environment, and all analyses were subject to formal verification procedures.

## Results

### Subject Characteristics

**Figure 1:**
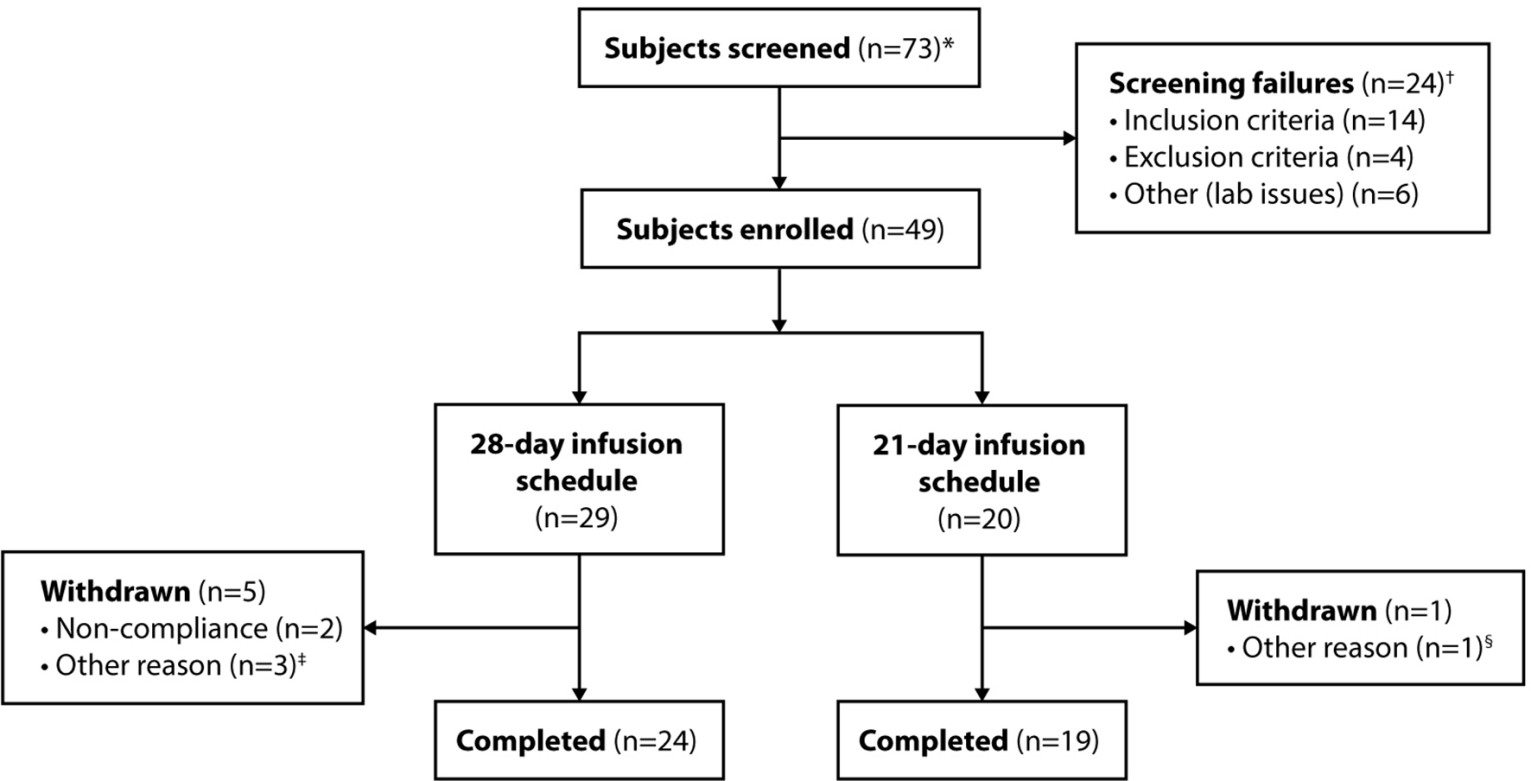
Subject disposition. *Includes subjects who were re-screened. ^†^Six of the 24 screen failures (5 subjects) were subsequently re-screened and enrolled in the study. ^‡^Withdrawal of consent (n = 2); withdrawal by sponsor due to melanoma diagnosis (n = 1). ^§^Inability to obtain IV access.

Of 73 screening assessments performed, 49 subjects were enrolled, treated with at least 1 dose of GC5107, and included in the ITT population (**Figure 1**). A total of 6 subjects withdrew from the study: 2 due to noncompliance, 1 due to the inability to obtain IV access, and 2 due to withdrawal of consent for reasons unrelated to GC5107. One subject in the 28-day infusion group was withdrawn by the sponsor due to a melanoma diagnosis. No subject withdrew due to an AE. A total of 43 subjects completed the study.

Subject demographics are shown in **Table 1**. Twenty-nine subjects received GC5107 on a 28-day schedule and 20 received GC5107 on a 21-day schedule. The total patient population included 33 adults aged 17 years or older, 8 adolescents aged 12 to 16 years and 8 pediatric subjects aged 2 to 11 years. The mean age of participants was 37.1 years (range, 3 to 70 years). The study population was predominantly white (95.9%) and non-Hispanic (91.8%), with males representing 57.1%.

**Table 1:** Subject demographics.

	28-day infusion (n = 29)	21-day infusion (n = 20)	Total (n = 49)
**Age at screening,** y			
Mean ± SD	41.5 ± 21.7	30.7 ± 23.2	37.1 ± 22.7
Median (Min, Max)	46.0 (7, 70)	18.5 (3, 69)	38.0 (3, 70)
**Age group,** n (%)			
2-11 y	4 (13.8)	4 (20.0)	8 (16.3)
12-16 y	2 (6.9)	6 (30.0)	8 (16.3)
17 y and older	23 (79.3)	10 (50.0)	33 (67.3)
**Gender,** n (%)			
Male	16 (55.2)	12 (60.0)	28 (57.1)
Female	13 (44.8)	8 (40.0)	21 (42.9)
**Race,** n (%)			
White	28 (96.6)	19 (95.0)	47 (95.9)
Other*	1 (3.4)	1 (5.0)	2 (4.1)
**Ethnicity**, n (%)			
Hispanic/Latino	2 (6.9)	2 (10.0)	4 (8.2)
Not Hispanic/Latino	27 (93.1)	18 (90.0)	45 (91.8)
**Geographic region,** n (%)			
US	18 (62.1)	18 (90.0)	36 (73.5)
Canada	11 (37.9)	2 (10.0)	13 (26.5)

*White/Lebanese (n=1); White/African American (n=1).

Max, maximum; Min, minimum; SD, standard deviation.

IVIG therapy baseline characteristics at the time of study enrollment are shown in **Table 2**. Mean disease duration since the first lifetime IVIG infusion was approximately 8.8 years. All subjects had been receiving IVIG infusions at regular 21- or 28-day intervals prior to enrollment at a dose level between 319 and 826 mg/kg (mean ± SD 538.5 ± 120.6 mg/kg). Subjects on a 28-day schedule had a lower mean IVIG dose prior to study entry than those on a 21-day schedule (501.2 *vs* 592.7 mg/kg, respectively). Trough IgG levels during most recent IVIG therapy prior to enrollment were above 500 mg/dL in all subjects (range, 521 to 1286 mg/dL), with no notable differences between the 2 infusion schedules.

**Table 2:** Subject history of IVIG therapy at baseline.

	28-day infusion (n = 29)	21-day infusion (n = 20)	Total (n = 49)
**Duration since first IVIG infusion,** y*			
n (missing)	28 (1)	20 (0)	48 (1)
Mean ± SD	8.8 ± 8.3	8.8 ± 6.7	8.8 ± 7.6
Median (Min, Max)	7.1 (0.2, 27.3)	9.3 (0.1, 18.8)	8.5 (0.1, 27.3)
**IVIG dose prior to enrollment,** mg/kg			
n (missing)	29 (0)	20 (0)	49 (0)
Mean ± SD	501.2 ± 94.0	592.7 ± 135.9	538.5 ± 120.6
Median (Min, Max)	497.0 (319, 693)	572.5 (324, 826)	520.0 (319, 826)
**IgG level prior to first lifetime IVIG infusion,** mg/dL			
n (missing)	23 (6)	17 (3)	40 (9)
Mean ± SD	393.6 ± 166.8	335.4 ± 202.3	368.9 ± 182.6
Median (Min, Max)	457 (10, 628)	329 (6, 679)	394 (6, 679)
**Trough IgG level during most recent IVIG therapy,** (mg/dL)			
n (missing)	29 (0)	20 (0)	49 (0)
Mean ± SD	930 ± 186.8	941.7 ± 195.5	934.8 ± 188.5
Median (Min, Max)	947 (521, 1286)	932 (563, 1280)	945 (521, 1286)

*Duration since first lifetime IVIG infusion (years)=(screening date – first lifetime IVIG infusion date + 1)/365.25.

IgG, immunoglobulin G; IVIG, intravenous immune globulin; Max, maximum; Min, minimum; SD, standard deviation.

### Efficacy

During the study period, comprising a total of 667 infusions representing 45.86 cumulative patient-years of treatment, a single primary endpoint event of aSBI occurred in the ITT population. This resulted in an incidence of acute SBIs of 0.02 events per patient-year, with an upper 99% CI limit of 0.21. The single case of bacterial pneumonia occurred in a 59-year-old male in the 28-day schedule group with a pre-enrollment history of recurrent pneumonia, chronic cough, shortness of breath, bronchiectasis, and asthma. The subject was admitted to the hospital, diagnosed with multi-segmental bronchopneumonia, and treated with antibiotics and corticosteroids. He was discharged after 3 days and the pneumonia resolved 7 days later.

A total of 38 subjects (77.6%) experienced a total of 135 infections other than aSBIs over the study period, resulting in a mean incidence of 2.9 infections/patient-year (**Table 3**). The mean annualized incidence of infections was higher in the 21-day than the 28-day schedule group (3.6 *vs* 2.4 infections/patient-year, respectively).

**Table 3:** Infections other than aSBI*, intent-to-treat (ITT) population.

	28-day infusion (n = 29)	21-day infusion (n = 20)	Total (n = 49)
**Infections other than aSBI**, n	64	71	135
** No. subjects with at least 1 infection,** n (%)	21 (72.4)	17 (85.0)	38 (77.6)
**Incidence,** number/patient-year			
Mean ± SD	2.4 ± 1.9	3.6 ± 2.9	2.9 ± 2.5
Median (Min, Max)	2.0 (0, 8.0)	3.5 (0, 12.2)	2.0 (0. 12.2)

*Infections defined as treatment-emergent adverse events (TEAEs) coded to MedDRA System Organ Class “Infections and infestations,” or otherwise suggestive of an infection by medical review.

aSBI, acute serious bacterial infection; Max, maximum; Min, minimum; SD, standard deviation.

Infections experienced by more than 5% of subjects during the study period are listed in **Table 4**. The most common infections were sinusitis, nasopharyngitis, upper respiratory tract infection, influenza, bronchitis, acute sinusitis, and urinary tract infection.

**Table 4:** Infections observed, ITT population.

	No. Subjects (%)	No. Events
**Total**	38 (77.6)	136
Sinusitis	13 (26.5)	17
Nasopharyngitis	6 (12.2)	12
Upper respiratory tract infection	6 (12.2)	10
Influenza	6 (12.2)	7
Bronchitis	6 (12.2)	6
Acute sinusitis	5 (10.2)	6
Urinary tract infection	5 (10.2)	5
Viral infection	4 (8.2)	4
Gastroenteritis viral	3 (6.1)	3
Otitis media	3 (6.1)	3
Viral upper respiratory tract infection	3 (6.1)	3

The proportion of subjects (38.8%) with trough IgG levels equal to or above the group mean trough (775.2 mg/dL) *vs* those below the group mean who experienced an infection other than aSBI was the same. However, having an IgG trough level equal to or above the group mean was associated with a higher number of infections than having an IgG trough level below the group mean (78 *vs* 57 infections, respectively). Therefore, the annual rate of infections was higher in the higher trough level group (3.3 ± 2.8 *vs* 2.6 ± 2.0 infections/patient-year, respectively).

Infection-related secondary endpoints are found in **Table 5**. Twenty-five subjects (51%) in the ITT population missed at least 1 day of work, school, kindergarten, daycare or were unable to perform normal activities due to infections. The number of days missed averaged 7.1 and ranged from 0 to 96. A total of 32 subjects (65.3%) had an unscheduled physician visit due to an infection during the study period, with a mean number of unscheduled physician visits of 2.3 days (range, 0 to 24 days). Two subjects (4.1%) were hospitalized due to an infection during the study, 1 for 2 days and 1 for 3 days, resulting in a mean duration of hospitalizations of 0.1 days. During the study period, 1 patient received IV antibiotics for a total of 3 days for the treatment of bacterial pneumonia. A total of 29 subjects (59.2%) received oral antibiotics during the study period for an average of 13.2 days (range, 0 to 127 days).

**Table 5:** Infection-related endpoints, ITT population.

Events due to infections	Subjects with at least 1 event [n (%)]	Days (Mean ± SD) (n=49)	Days (Min, Max) (n = 49)
Days missed from work/school/daycare or unable to perform daily activities	25 (51)	7.1 ± 18.04	0, 96
Days of unscheduled visits to physicians	32 (65.3)	2.3 ± 3.75	0, 24
Days of hospitalizations	2 (4.1)	0.1 ± 0.53	0, 3
Yearly hospitalization rate*		0.1 ± 0.31	0, 2
Yearly hospitalization duration^†^		0.1 ± 0.51	0, 3
Days of antibiotic use^‡^			
IV	1 (2.0)	0.1 ± 0.44	0, 3
Oral	29 (59.2)	13.2 ± 22.09	0, 127

*Yearly Hospitalization Rate=number of hospitalization/subject follow-up time in years.

^†^Yearly Hospitalization Duration=total hospitalization duration in days/subject follow-up time in years.

^‡^Based on the total number of days of antibiotic use for each subject. Mean and SD are calculated based on weighting for the duration of data available for each subject. IV, intravenous; Max, maximum; Min, minimum; SD, Standard deviation.

### Safety

Of 29 subjects treated according to the 28-day schedule, 24 (82.8%) received all 13 infusions prescribed by the protocol, with the remaining 5 subjects receiving between 1 and 11 infusions. Of 20 subjects treated according to the 21-day schedule, 19 (95%) received all 17 protocol-prescribed infusions, with 1 subject receiving 9 infusions. In total, there were 667 infusions among the 49 participating subjects over the 12-month study period comprising 45.86 cumulative patient-years of treatment.

In the total ITT population (n=49), the proportion of infusions with TAAEs occurring during or within 72 hours after an infusion of GC5107 was 0.24, with an upper 95% CI limit of 0.31 (**Table 6**).

**Table 6:** Proportion of infusions with TAAEs,* ITT population.

	28-day infusion (n = 29)	21-day infusion (n = 20)	Total (n = 49)
Total no. infusions	335	332	667
No. infusions with TAAEs	96	53	149
Proportion of infusions with TAAEs	0.31	0.16	0.24
(upper 95% CI limit)^†^	(0.40)	(0.22)	(0.31)

*TAAE, Temporally associated adverse event occurring during or within 72 hours following infusion of GC5107, whether or not it was thought to be caused by GC5107.

^†^Upper one-sided 95% confidence interval (CI) limit.

A summary of TAAEs experienced by more than 5% of subjects in the ITT population is found in **Table 7**. The most common included headache (experienced by 49% of subjects), nausea (24.5%), fatigue (18.4%), nasal congestion (10.2%), cough, and diarrhea (8.2% each).

**Table 7:** Most common TAAEs* (experienced by >5% of subjects), ITT population.

Total number TAAEs	By patient (n = 49)		By infusion (n = 667)	
	241		241	
	n	%	n	%
Number of subjects/infusions with TAAEs	42	85.7	149	22.3
Headache	24	49.0	68	10.2
Nausea	12	24.5	20	3.0
Fatigue	9	18.4	29	4.3
Nasal congestion	5	10.2	5	0.7
Cough	4	8.2	5	0.7
Diarrhea	4	8.2	4	0.6
Arthralgia	3	6.1	4	0.6
Pyrexia	3	6.1	4	0.6
Sinus congestion	3	6.1	4	0.6
Abdominal pain upper	3	6.1	3	0.4
Infusion site extravasation	3	6.1	3	0.4
Pain	3	6.1	3	0.4
Oropharyngeal pain	3	6.1	3	0.4
Urticaria	3	6.1	3	0.4

*TAAE, Temporally associated adverse event occurring during or within 72 hours following infusion of GC5107, whether or not it was thought to be caused by GC5107, by MedDRA preferred term.

A summary of additional safety results is found in **Table 8**. All 49 subjects experienced at least 1 AE during the 12-month treatment and follow-up period. The majority of subjects (n=42, 85.7%) experienced at least 1 TAAE that occurred during or within 72 hours after an infusion. There were no notable differences between the 2 infusion schedules in the proportion of subjects who experienced a TAAE; however, the number of infusions with TAAEs was considerably higher in the 28-day compared to the 21-day schedule group (**Table 6**).

**Table 8:** Summary of adverse events, ITT population.

	28-day infusion (n = 29)		21-day infusion (n = 20)		Total (n = 49)	
	Subjects, n (%)	Events, n	Subjects, n (%)	Events, n	Subjects, n (%)	Events, n
Frequency of all AEs	29 (100)	492	20 (100)	251	49 (100)	743
Number of TAAEs*	25 (86.2)	169	17 (85.0)	72	42 (85.7)	241
Suspected ARs^†^	14 (48.3)	84	12 (60.0)	29	26 (53.1)	113
Severe AEs	6 (20.7)	11	1 (5.0)	1	7 (14.3)	12
Treatment-emergent serious AEs (SAEs)	3 (10.3)	4	1(5.0)	1	4 (8.2)	5
AEs leading to infusion rate decrease	1 (3.4)	1	0	0	1 (2.0)	1
AEs leading to dose interruption	2 (6.9)	5	3 (15.0)	3	5 (10.2)	8
AEs leading to withdrawal or early discontinuation from the study	0	0	0	0	0	0

*TAAE, Temporally associated adverse event occurring during or within 72 hours following infusion of GC5107, whether or not it was thought to be caused by GC5107.

AE, adverse event; SAE, serious adverse event.

^†^AR, adverse reaction, or AE determined to be at least possibly related to GC5107.

Twenty-six subjects (53.1%) experienced 113 AEs assessed by the investigator as at least possibly related to GC5107 (listed as suspected ARs in **Table 8**). Headache (15 subjects, 30.6%), fatigue, and nausea (7 subjects, 14.3% each) were the most commonly reported. Two subjects experienced 1 AE each (moderate headache and moderate urticaria) that were assessed to be certainly related to GC5107. Both events occurred during or within 1 hour of infusion.

Overall, the majority of AEs reported during the study (588/743, or 79.1%) were mild in intensity. Seven subjects (14.3%) experienced a total of 12 severe AEs. Three of the severe AEs were assessed by the investigator as at least possibly related to GC5107 and all 3 occurred in 1 patient in the 28-day schedule group (2 events of headache and 1 event of nausea).

Four subjects (8.2%) experienced a total of 5 treatment-emergent serious AEs (SAEs) during the study. Of these, 1 was assessed by the investigator as moderate (influenza), and 4 were assessed as severe in intensity (urticaria, bacterial pneumonia, acute myocardial infarction, and squamous cell carcinoma of the tongue). None of the SAEs were deemed by the investigators as related to GC5107.

The infusion rate was decreased due to an AE in 1 patient (**Table 8**). The event (headache) occurred at an infusion rate of 8 mg/kg/min, was assessed by the investigator as moderate and certainly related to GC5107, and resolved within 2 hours. A total of 5 subjects (10.2%) experienced 8 AEs leading to dose interruption. Of these, 1 AE (infusion site extravasation) occurred in 3 subjects (6.1%); the remaining events (1 event of heart rate increased, 3 events of infusion site swelling, and 1 event of nausea) occurred in 1 patient each. Seven of the 8 AEs leading to dose interruption were determined by investigators as not related to GC5107, and 1 (heart rate increased) was assessed as possibly related. All 8 AEs leading to dose interruption were assessed as mild. There were no AEs leading to death or study discontinuation.

Analysis of clinical and laboratory safety parameters revealed no clinically meaningful changes in hematology, clinical chemistry, urinalysis, or vital signs parameters, or weight measurements. A shift from negative to positive hepatitis B virus (HBV) DNA (by nucleic acid testing) occurred in 2 subjects. Both tested negative for HBV DNA at the subsequent assessment. No other changes occurred in viral safety parameters during the study.

A positive direct Coombs test was detected at least once during the study in 17 subjects (34.7%); 2 of these subjects also tested positive prior to the first GC5107 infusion. None of the 17 subjects had any laboratory findings or symptoms suggestive of hemolysis. Two subjects had Coombs test abnormalities deemed clinically significant that were reported as AEs – both events were assessed by the investigator as mild and not related to GC5107. No AEs indicative of hemolysis were reported.

### Pharmacokinetics

A total of 27 subjects (15 in the 28-day schedule group and 12 in the 21-day schedule group) were included in the PK subpopulation and contributed samples for analysis of serum concentrations of total IgG and IgG subclasses after the 5^th^ infusion of GC5107.

A summary of key PK parameters of total IgG (uncorrected values) following the 5^th^ infusion is shown in **Table 9**. Of note, there were only a few subjects with reliable estimates (3 subjects in the 28-day and 2 subjects in the 21-day schedule group) for Vss and CL (**Table 9**), thus results for these parameters should be evaluated with caution.

**Table 9:** Pharmacokinetics of total IgG* PK population.

	28-day infusion	21-day infusion	Total
	Mean ± SD	Mean ± SD	Mean ± SD
	(Min, Max)	(Min, Max)	(Min, Max)
**C_max_,** mg/dL	n=15	n=11	n=26
	2122 ± 350.3	2245 ± 406.0	2174 ± 372.2
	(1501, 2668)	(1601, 3129)	(1501, 3129)
**AUC_0-t_**, day•mg/dL	n=15	n=11	n=26
	31490 ± 7249.6	26470 ± 4197.3	29370 ± 6548.2
	(25270, 52520)	(18460, 34320)	(18460, 52520)
**T_max_,** days	n=15	n=12	n=27
	0.25 ± 0.34	0.70 ± 0.99	0.45 ± 0.73
	(0.08, 1.08)	(0.07, 3.11)	(0.07, 3.11)
**T_1/2_,** days	n=11	n=10	n=21
	29.6 ± 11.0	29.0 ± 25.3	29.29 ± 18.67
	(13.2, 51.5)	(13.1, 99.8)	(13.1, 99.8)
**VSS,** mg/kg	n=3	n=2	n=5
	31.5 ± 6.3	41.6 ± 0.11	35.5 ± 7.1
	(24.2, 35.7)	(41.5 41.6)	(24.2, 41.6)
**CL,** mL/day/kg	n=3	n=2	n=5
	1.26 ± 0.60	2.04 ± 0.19	1.57 ± 0.61
	(0.65, 1.84)	(1.90, 2.17)	(0.65, 2.17)

*Not corrected for baseline IgG levels.

AUC, area under the (time concentration) curve; C_max_, maximum serum concentration; CL, clearance; Max, maximum; Min, minimum; SD, standard deviation; T_1/2_, elimination half-life_;_ T_max_, time to maximum serum concentration; V_ss_, apparent volume of distribution at steady state.

There were 18 instances of trough IgG levels below the 500 mg/dL target in 12 subjects (3 in the 21-day schedule group and 9 in the 28-day schedule group). Of the 12 subjects, there were 5 children (aged 3 to 11 years), 2 adolescents (both aged 15 years), and 5 adults (aged 22 to 69 years). The majority of subjects (10/12, 83.3%) with trough IgG levels below 500 mg/dL were males.

Mean trough levels of anti-*H influenzae* type b, anti-*S pneumoniae*, and anti-tetanus toxoid antibodies remained comparable to pre-treatment values throughout the treatment period in both infusion schedule groups. All subjects tested positive for anti-CMV IgG antibodies at each assessment time point.

Observed IgG subclass levels after the 5^th^ infusion were consistent with a physiologic distribution pattern. For all IgG subclasses, mean concentrations were generally higher, and mean half-life shorter, in the 21-day compared to the 28-day schedule groups.

## Discussion

This phase 3 study was designed to evaluate the safety, efficacy, and pharmacokinetics of GC5107, a new 10% liquid IVIG product developed by GC Pharma, in subjects with PIDs. The study was designed in accordance with current FDA guidance for clinical trials of IVIG products in support of their marketing as replacement therapy for PIDs (12).

Primary safety and efficacy analyses were based on the ITT population, consisting of all subjects enrolled in the study who received any amount of GC5107. Forty to 50 subjects are considered sufficient to power a study to demonstrate a rate of aSBI of less than 1 per subject-year and establish efficacy of an IVIG product for the treatment of PIDs (12).

A planned sample size of at least 42 subjects was estimated to provide approximately 80% power to reject the null hypothesis of aSBI incidence ≥1.0 per patient-year, provided that the true rate of aSBIs in the study population was below 0.56 per patient-year under the Poisson assumption. To guard against dropout, up to 50 subjects were planned to be enrolled. A noninferiority test was applied for the primary efficacy endpoint.

Over the 12 months of this study, 1 of 49 (2%) of subjects in the ITT population experienced an aSBI during a total cumulative follow-up of 45.86 patient-years. This reflects an overall rate of 0.02 aSBI per patient-year (upper 99% CI limit: 0.21), well below the prespecified endpoint. The efficacy of GC5107 reported here for preventing infections in PID patients is comparable to published results from similarly designed studies of other IG products currently marketed in the US (13–24).

The mean incidence of infections other than aSBIs reported here was 2.9 infections/patient-year. This incidence, too, compares favorably with that reported in studies of other IG products (14–16, 18–21, 25, 26). The incidence of infections other than aSBI was noticeably higher in the 21- *vs* 28-day infusion schedule groups (3.6 ± 2.9 *vs* 2.4 ± 1.9 infections/patient-year, respectively, **Table 3**) – despite the 21-day schedule group having received higher doses of IVIG and experiencing higher IgG trough levels prior to study enrollment (**Table 2**). One explanation for this finding may be that subjects in the 21-day schedule group had been treated with higher doses of IVIG prior to study enrollment due to a history of more frequent infections. End-organ damage to sinuses and airways as a result of chronic infections may have made this group more likely to experience infections during this study.

Compared to subjects with IgG trough levels below the group mean, those with IgG trough levels equal to or above the group mean had a higher annual rate of infections (3.3 ± 2.8 *vs* 2.6 ± 2.0 infections/patient-year, respectively). Factors that may have contributed to this unexpected result include a longer follow-up time (by approximately 2.1 patient-years) and greater disease severity, requiring higher IVIG doses among subjects with equal or higher trough IgG levels than the group mean.

The efficacy of GC5107 was also demonstrated by the low rates of IV therapeutic antibiotic use (mean, 0.1 days), oral therapeutic antibiotic use (mean, 13.2 days), unscheduled physician visits (mean, 2.3 visits), and rate of missed work/school/daycare days (mean, 7.1 days). During the study, 2 subjects (4.1%) required hospitalization due to an infection. These results compare favorably to those reported in studies of other IG products for the treatment of PID (15, 16, 18–20).

Demonstration of adequate safety of IVIG products requires that the upper 95% CI limit of the probability that an infusion is associated with an AE is below 0.40 (12). The current study met the primary safety endpoint, as the proportion of infusions with TAAEs occurring during or within 72 hours after the infusion was 0.24 (upper 95% CI: 0.31) (**Table 6**). The overall frequency and types of AEs reported in the current study are consistent with the known safety profile of other IG products.

The proportion of infusions with TAAEs was higher in the 28-day compared to the 21-day infusion schedule group. It should be noted that 86 of 169 (50.9%) of the TAAEs reported in the 28-day schedule group were reported by 4 of the 29 subjects (13.8%). Due to the relatively low number of subjects per infusion schedule group, this finding should be interpreted with caution. Viral safety was confirmed by lack of viral DNA/RNA 3 to 4 weeks after the last infusion of GC5107.

During treatment with GC5107, the range of mean trough IgG concentrations was higher in the 21-day (762.7 mg/dL to 966.0 mg/dL) compared to the 28-day infusion schedule groups (708.3 to 768.1 mg/dL). Mean trough IgG levels are typically above 900 mg/dL in subjects treated with available IVIG products according to the 21-day schedule and above 800 mg/kg in subjects receiving IVIG treatment every 28 days (6). However, in this study, lower mean trough IgG levels were detected at baseline (820.3 mg/dL for the 21-day infusion schedule group and 726.7 mg/dL for the 28-day infusion schedule) and maintained throughout the study. Importantly, these somewhat lower trough IgG levels did not translate to diminished efficacy, as evidenced from the analyses of the primary and all secondary efficacy endpoints.

During this study 12 subjects (3 in the 21-day schedule group and 9 in the 28-day schedule group) experienced IgG trough levels below the 500 mg/dL target. Circumstances that may have contributed to this included longer intervals between infusions (between 33 and 36 days, and 35 days, respectively, in 2 subjects in the 28-day schedule group), recent infections (1 subject in each infusion schedule group), and increases in body weight in 2 pediatric subjects (1 in each infusion schedule group). Importantly, none of the 12 subjects experienced a serious infection, and only 1 experienced a non-serious infection (sinus infection) within 4 weeks after a below-target trough IgG measurement.

After infusion of GC5107, mean serum IgG half-life was approximately 30 and 29 days for the 28-day and 21-day infusion schedule groups, respectively. Other PK characteristics of GC5107 were comparable to those reported for other 10% IVIG products (**Table 9**).

In conclusion, 1 year of treatment with GC5107 was effective in preventing infections in subjects with PID and was well tolerated, with no SAEs attributed to GC5107 and no AEs leading to withdrawal. The results of this phase 3 study demonstrate that GC5107 is a safe and effective treatment for the prevention of infections in patients with PID.

## Data Availability Statement

The original contributions presented in the study are included in the article. Further inquiries can be directed to the corresponding author.

## Ethics Statement

The studies involving human participants were reviewed and approved by Advarra or by local Institutional Review Boards (IRBs). Written informed consent to participate in this study was provided by the participants’ legal guardian/next of kin.

## Author Contributions

RW contributed substantially to data interpretation, manuscript writing and editing. All authors contributed to the article and approved the submitted version.

## Funding

This study was funded by GC Pharma, Korea.

## Conflict of Interest

AE has participated in advisory boards for ALK Abello, AstraZeneca, Aralez, Bausch Health, LEO Pharma, Merck, Novartis, and Pfizer; has served as a speaker for ALK Abello, The ACADEMY, Aralez, AstraZeneca, Medexus, and Mylan; and has been a consultant to Bayer LLC and Regeneron. Her institution has received research grants from ALK Abello, Aralez, AstraZeneca, Bayer LLC, GC Pharma, Medexus, Novartis, Sanofi and Regeneron. WL has served as a consultant for Accordant, BioCryst, Biomarin, CSL Behring, Express Scripts, Fresenius Kabi, Intellia, Kalvista, Magellan, Optum, Pharming, Pharvaris, and Shire/Takeda; serves as a speaker for BioCryst, CSL Behring, Pharming, Shire/Takeda, Grifols, Astra Zeneca, Sanofi/Regeneron and GSK; and receives grants or research support from ALK, BioCryst, CSL Behring, Gossamer, GC Pharma, Grifols, Ionis, Kalvista, Kedrion, Shire/Takeda and Therapure. He is a member of the US Hereditary Angioedema Association Medical Advisory Board. RW serves as an investigator for CSL Behring, Grifols, Kedrion, GC Pharma, Takeda, and TherapureBio; serves as a consultant for ADMA Biologicals, Grifols, GC Pharma, Takeda, and TherapureBio; and serves as a speaker for CSL Behring, Grifols, and Takeda.

The remaining authors declare that the research was conducted in the absence of any commercial or financial relationships that could be construed as a potential conflict of interest.

The authors declare that this study received funding from GC Pharma, Korea. The funder was involved in the study design, analysis and interpretation of data, review of the manuscript, and the decision to submit the manuscript for publication.
